# Effectiveness of “Escape Room” Educational Technology in Nurses’ Education: A Systematic Review

**DOI:** 10.3390/nursrep14020091

**Published:** 2024-05-13

**Authors:** Héctor González-de la Torre, María-Naira Hernández-De Luis, Sergio Mies-Padilla, Rafaela Camacho-Bejarano, José Verdú-Soriano, Claudio-Alberto Rodríguez-Suárez

**Affiliations:** 1 Research Support Unit, Insular Maternal and Child University Hospital Complex, Canary Health Service, 35016 Las Palmas de Gran Canaria, Spain; 2 Nursing Department, Faculty of Healthcare Science, Universidad de Las Palmas de Gran Canaria (ULPGC), 35016 Las Palmas de Gran Canaria, Spain; 3 Las Remudas Primary Health Care Centre, Canary Health Service, 35213 Las Palmas de Gran Canaria, Spain; mherluif@gobiernodecanarias.org; 4 Department of Obstetrics and Gynaecology, Insular Maternal and Child University Hospital Complex, Canary Health Service, 35016 Las Palmas de Gran Canaria, Spain; sergio.mies101@alu.ulpgc.es; 5 Department of Nursing, University of Huelva, 21007 Huelva, Spain; rafaela.camacho@denf.uhu.es; 6 Department of Community Nursing, Preventive Medicine, Public Health and History of Science, Faculty of Health Sciences, University of Alicante (UA), 03690 Alicante, Spain; pepe.verdu@ua.es

**Keywords:** education, nursing, educational technology, gamification, systematic review

## Abstract

Escape room games are educational gamification technologies that consist of introducing a team of players into a physical or digital space in search of clues to answer puzzles, riddles or enigmas and solve a mystery or problem. This study aims to determine the effectiveness of escape room games on the training of nursing students in an international context. A systematic review was carried out in MEDLINE, WOS, SCOPUS, CINAHL and LILACS databases using the MeSH terms “Education, Nursing” and “Educational Technology”, and the free term “Escape room”, combined with Boolean operators AND/OR. Intervention studies in Spanish, English and Portuguese were included, without limitation for the year of publication. Selection and critical appraisal were conducted by two independent reviewers. A total of *n* = 13 interventional studies were included (*n* = 2 Randomized Clinical Trials and *n* = 11 quasi-experimental design). Escape rooms are a recent and growing educational methodology, increasingly used in academia and in the training of nurses and nursing students. However, it is necessary to expand their use and the quality of the studies in a greater number of contexts. Furthermore, it is necessary to homogenize and standardize validated instruments to evaluate the effectiveness of escape rooms in the nursing education area.

## 1. Introduction

The use of educational technologies aims to facilitate and improve learning through the creation, use and management of appropriate technological processes and resources [1]. These educational technologies should facilitate collaboration among students, stimulate student problem solving and seek an “authentic approach”, improving their motivation and engagement [1,2]. This is why the search for more effective educational technologies has aroused great interest in the educational community [3,4]. This is especially applicable in the case of the education of health sciences students in general and nursing students in particular [1,4].

One of the educational technologies that has piqued the most interest in recent years has been gamification [4,5]. Although there is no uniform definition regarding this term [5], we can say that gamification includes the use of various game elements in the academic setting with the aim of improving the academic learning performance and motivation of students [5,6]. These game elements should be interpreted widely, as they can include different techniques and methods [1,5,6], but always with the main purpose of using them to achieve a didactic and educational objective that should be clear and well defined [5]. Therefore, the main purpose should never be entertainment, but to improve students’ learning of a specific subject or area, as well as to help in the acquisition of certain clinical-practical skills or competencies [5,7].

One of the educational techniques that have been included in gamification is the so-called “escape rooms” (ERs) [4,8]. ER games consist of introducing a team of players in a physical or digital space in search of clues to complete puzzles, riddles or enigmas, with the aim of solving a mystery or a problem. ER games have the aim of acquiring professional skills in a complementary way to other teaching methods [8,9]. ER games have been used in recent years in the field of health sciences education, including nursing studies, whether undergraduate or graduate [4,8,10].

Recently, Reinkemeyer et al. have examined the use of ER games in nursing, concluding that they are effective in improving nurses’ knowledge on different topics [10]. According to these authors, the ER games were organized around four main narrative themes: group dynamics, training, theoretical aspects and identified barriers. However, this study did not perform statistical data extraction reporting on the effectiveness of ER games and did not undertake a joint analysis of the results. Thus, this review included only studies published in English. A new systematic review of this topic in other international contexts was proposed with the aim of evaluating the effectiveness of ER games in the specific training of nursing students based on the following review question: what is the effectiveness of the use of ER games as an educational technology for training nursing students at international context? Therefore, the aim of this review was to determine the effectiveness of ER games on the education of nursing students in the international context.

## 2. Materials and Methods

Design: A systematic review was carried out according to the methodology of the Joanna Briggs Institute (JBI) [11]. The report of the results followed the Preferred Reporting Items for Systematic Review and Meta-Analysis (PRISMA) Statement criteria [12]. The review protocol has been registered in PROSPERO under number CRD4202222374207. As this review is on the effectiveness of an intervention, the research question has been shaped using the following structure: Population (P), Intervention (I), Comparison (C) and Outcomes (O) [13], with P being undergraduate nursing students, I being ER games with physical or digital approaches, C being other gamification games or traditional educational techniques, and O being knowledge, satisfaction and attitudes with the training received.

Sources of information: The first step was to identify previous publications on the topic of interest through various searches in PROSPERO and Google Scholar^®^ databases that could answer the search question. After this initial check, searches were conducted in December 2023 in the following Health Sciences databases: MEDLINE (PubMed), MEDLINE (OVID), SCI Expanded (Web of Science), SCOPUS (SCOPUS-Elsevier) and CINAHL (EbscoHOST).

Search strategies: The DeCS/MeSH descriptors “Education, Nursing” and “Educational Technology” were used, as well as the free term “Escape room” using Boolean operators AND/OR. Where appropriate, methodological filters were applied. The searches were piloted in PubMed. The search process was developed by one of the researchers (C.-A.R.-S.) and verified by a second researcher (H.G.-d.l.T) using the PRISMA-S for searching extension [14]. All references were exported to Mendeley Reference Manager Online^®^ for screening. Table 1 shows the search strategies performed in each of the databases.

Inclusion criteria: Studies published up to December 2023 in Spanish, English or Portuguese that have addressed the use of ER games in the context of undergraduate education in nursing students were included. Only experimental intervention studies were included: randomized clinical trials (RCTs) and quasi-experimental studies (pre-post designs with or without a control group). No time limit was set for the year of publication.

Exclusion criteria: Studies conducted on graduate nurses, and other gamification games or traditional educational techniques were excluded. Other review studies (systematic, exploratory or narrative), studies with quantitative observational, analytical and descriptive designs, case studies and qualitative designs with any methodology were excluded. Publications that did not correspond to research studies (such as editorials and letters to the editor) were also excluded. Gray literature was not included.

Selection and classification of studies: After performing the searches, duplicate records were eliminated and screened by title and abstract. The full-text documents of the selected records were then retrieved to assess their eligibility according to inclusion and exclusion criteria. Screening was performed by peer review (H.G.-d.l.T. and S.M.-P.) and, in case of discrepancies, a third researcher decided (C.-A.R.-S.).

Definition of the study variables: Bibliometric variables on the affiliation of studies, as well as variables on the statistical results of the studies have been extracted. The main research outcome corresponded to the knowledge, and secondary outcomes were satisfaction and attitudes with the training research. However, knowledge, satisfaction and attitudes have been extracted from all studies, regardless of whether they were primary or secondary results. Additionally, other primary or secondary outcomes not included that have been reported in the different studies have also been extracted.

Evaluation and data extraction: Studies identified as potentially eligible for inclusion were distributed for peer review by two investigators (J.V.-S. and R.C.-B.) and discrepancies were resolved by a third researcher (M.-N.H.-D.L.). To assess the quality of the studies, the JBI critical appraisal tools appropriate to each research design were used, establishing as a criterion of good quality a score of more than 50% with respect to the items included in each tool (for RCT-13 items, a score ≥ 7 was considered good quality and for quasi-experimental studies—9 items, a score ≥ 5). Finally, the following information was extracted from the studies: country and year, design, main/secondary outcomes, instrument used to measure the effectiveness of ER games, characteristics of the ER games (type, setting and duration of the ER games sessions, size and composition of the groups) and the population in which it was performed. For continuous quantitative variables, statistical data on mean scores and standard deviations were extracted, and for qualitative variables, percentages and frequencies were extracted. The *p*-values were also extracted to test the hypothesis contrasts and the effect sizes when they were calculated. Data extraction was carried out independently by two researchers (H.G.-d.l.T. and S.M.-P.) and discrepancies were resolved by a third researcher (C.-A.R.-S.).

## 3. Results

**Figure 1:**
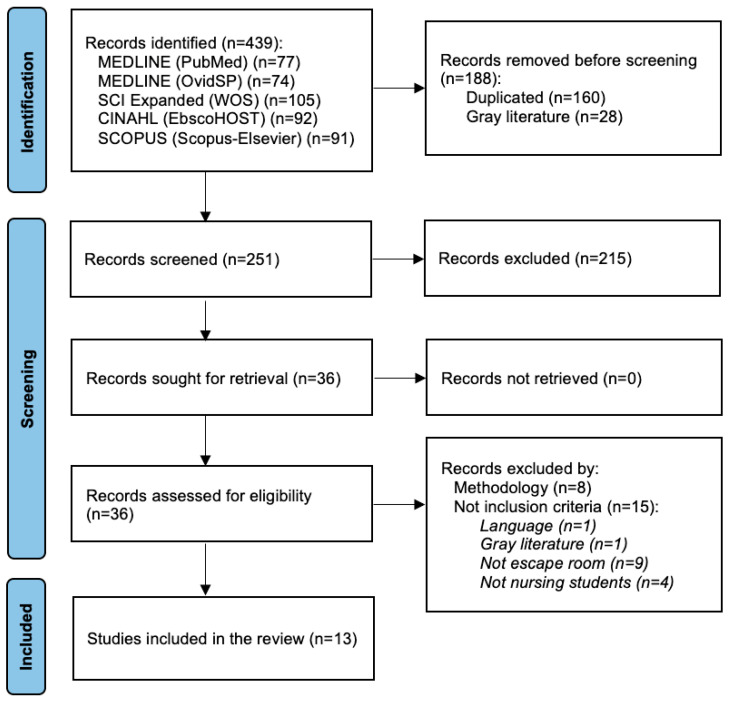
Flow diagram.

The number of records retrieved was *n* = 439; after eliminating duplicates (*n* = 160) and gray literature (*n* = 28), *n* = 251 records were screened by title and abstract. Of these, *n* = 215 records were excluded because they did not meet the inclusion criteria, while *n* = 36 records met the criteria for full-text evaluation. After the critical appraisal process, *n* = 13 studies were included in the review, as shown in the flow diagram in Figure 1.

In the full-text critical appraisal process, *n* = 8 studies were excluded for not meeting the minimum methodological quality and *n* = 15 were excluded for not meeting the inclusion criteria (Table S1). The critical appraisal process of the included studies is shown in Supplementary Table S2.

Regarding the methodological design of the studies, RCTs (*n* = 2) and quasi-experimental studies (*n* = 11) were included. The quasi-experimental studies consisted of different designs such as pre- and post-experimental with control group (*n* = 4), pre- and post-experimental (*n* = 6) and quasi-experimental with control group (*n* = 1).

Table 2 shows the year and country of publication, design, themes and learning topics, aim and main/secondary outcomes and the conclusions for each study.

The thematic areas covered by studies were very disparate: gerontology, interprofessional collaboration, maternity care, neurological disorders, anatomy, severe mental illness interprofessional education or interprofessional practice, clinical skills and cardiovascular critical care. Four studies addressed the subject of interprofessional collaboration, although from different perspectives (effective communication and teamwork, interprofessional management of opioid use disorder, improve teamwork and sepsis management, and post-operative precautions).

Regarding the design of the ER games, the educational activities were also heterogeneous in different studies, with physical settings (*n* = 8), virtual/online settings (*n* = 3) or mixed (physical and virtual) settings (*n* = 2). 

The clinical results of the studies are shown in Table 3. 

## 4. Discussion

As a result of the quick development and diffusion of gamification, an increasing number of studies and reviews are being published each year examining this educational methodology in healthcare workers [28,29]. Gamification is associated with positive perceptual, cognitive, behavioral, affective, and motivational effects and outcomes [29,30], as well as having the potential to offer learners the opportunity to engage in active learning, solve clinical problems, and acquire experience in a risk-free environment without the need to involve patients [30].

Within gamification, ER games have been rapidly growing in recent years [8,10,31]. In addition to the effects previously pointed out, this learning system constitutes a method able to decrease the generation gap that sometimes exists between students and teachers [27], being an example of educational technology that can help to overcome the dissonance between traditional methodologies and the needs of more innovative educational methodologies demanded by the new generations of students [32], all with a very acceptable economic cost [33,34]. This implies that systems capable of collecting the perceptions and experiences of the participants should always be included in the design of the ER games since in this way key information can be obtained to identify aspects that can be improved [8]. Therefore, debriefing is a necessary element to be included in ER games [8,35], with some authors going so far as to state that in healthcare simulation, “debriefing is just as or even more important than the simulation” [36]. Some of the studies included in this review included various debriefing systems for this purpose [19,22,25], although without uniformity regarding the method used for this purpose. Some authors such as Eukel and Morrell [8] and Eukel et al. [33] recommend using a survey of their design.

Similarly, it is also desirable to assess participant satisfaction with the activity [8,36]. However, many of the studies included in this review did not evaluate it or did so only superficially [22]. Only Gutiérrez-Puertas et al. used a validated tool, the Gameful Experience Scale (GAMEX), although the aim of their work was directly to understand the gameful experience and satisfaction of nursing students in the evaluation of their clinical skills [26]. The GAMEX is an instrument developed by Eppman et al. [37] that measures the gameful experience and is composed of 27 items divided into 6 dimensions: Enjoyment, Absorption, Creative thinking, Activation, Absence of negative affect and Dominance. The responses are answered on a Likert-type scale, with values from 1 (never) to 5 (always), and a total score can be calculated or by dimensions. A higher score indicates a more positive experience regarding the gaming experience. The results reported by the study of Gutiérrez-Puertas et al. indicate acceptable satisfaction for the ER games experience in their case [38], like other studies included in this review that reported high degrees of satisfaction [39]. 

Although GAMEX is not a specific instrument for ER games, we consider it advisable to use this tool to evaluate the students’ experience with respect to ER games, since in addition to being able to measure the participants’ satisfaction with the activity in an objective way, it allows us to compare this educational technology against other types of gamification [40]. One dimension of this scale even allows the detection of the presence of eventual negative effects in the gamification activity. Elevated anxiety levels have been reported in nursing students related to clinical laboratory practicums and simulations [41,42]. Although more research addressing how ER games affect students’ anxiety levels is needed [43], in the design of ER games it is always imperative to guarantee a sense of safety among participants [38]. 

The present study was designed to answer the guiding question of this review and was initially aimed at conducting a meta-analysis to evaluate the effectiveness of ER games as an educational technology specifically in nursing. As such, only studies of experimental design were exclusively included, unlike the recent review by Quek et al., which included studies of all types of designs [4]. However, the high clinical heterogeneity found did not allow a meta-analysis to be performed, being one of the main limitations of this review, although this aspect is not new and has already been pointed out. The Cochrane review on the effectiveness of gamification educational activities in health sciences personnel conducted by Akl et al. cannot perform this meta-analysis either due to the lack of methodologically robust studies [30]. Quek et al. were also unable to perform a meta-analysis, despite including studies with all types of healthcare students in their review [4]. Therefore, the most important aspect to highlight as a result of this review is the lack of uniformity and the enormous heterogeneity that exists between the various studies that have been carried out with ER games in nursing. This situation affects all the elements, from the study designs to the thematic areas, to the tools or instruments used in the evaluation of their effectiveness, but especially to the measure’s outcomes of the studies. Even in those cases where a similar main outcome variable was assessed (e.g., measure of knowledge), the disparity of the topics and themes discouraged the performance of meta-analysis. This aspect should be considered in future studies carried out with ER games; as far as possible, researchers should try to standardize the interventions to be able to carry out more global evaluations of this educational technology.

A particularly relevant aspect concerns the study designs. All the studies included in our review are quasi-experimental, except for two RCTs by Rodriguez-Ferrer et al. [21] and Fusco et al. [23]. Regarding the quasi-experimental studies, only five studies had a control group [15,17,20,24,26]. Therefore, a priority aspect that emerges from our results is the need to conduct RCTs that provide more solid evidence of the effectiveness of ER games as an educational technology. This is extensible both to ER games aimed at nursing students and other health sciences students [4,31]. 

The data extracted from the included studies and reported on were sectioned by a population of nursing students versus pharmacy, physical therapy or health science students. In contrast to some of the previously mentioned reviews [4,30,31], this review focused exclusively on nursing students. However, studies of ER games in graduated nurses were not included, so the usefulness of ER games in the continuing education of already graduated nurses still needs to be explored in future studies. In addition, in some cases, nursing students were integrated into groups where there were students from other disciplines or areas [16,18,22,23,24,25]. 

Interprofessional collaboration and education is precisely one of the thematic areas where the use of ER games has been most explored [4,16,43,44]. Four studies (Hursman et al. [18], Wettergreen et al. [22], Fusco et al. [23], Foltz-Ramos et al. [24]) focused on this topic. Gamification is often used to encourage team building in businesses [3,7,44], so it is logical to also use this new tool for interdisciplinary team building in healthcare professionals, especially in areas that require close professional cooperation [44,45]. ER games can provide work teams with several benefits, in addition to the inherent effect of clinical simulation itself, as communication skills among the professionals that make up the teams are especially improved [22,45,46,47]. 

Although these aspects are undoubtedly important and are sufficient reason to implement ER games in educational programs, we should not forget that the central objective of any educational technology or methodology is the transmission of knowledge. Most of the studies included in the review were primarily motivated by the need to improve participants’ knowledge of a specific subject area, either in a single group (with a before and after measurement) or by comparing two groups. All studies found statistically significant differences with respect to these improvements, which indicates ER games is useful for increasing participants’ level of knowledge, something that has been previously pointed out in the literature [4,7,31,48]. However, we would like to call attention to several aspects that we consider important. On the one hand, none of the included studies used a validated instrument for the measurement and evaluation of knowledge; they always used ad hoc questionnaires, which provided little information on the psychometric properties or reliability of the instrument. This is one reason that has contributed to impeding the performance of a meta-analysis. Future studies should try to improve the choice of measurement instruments used to assess knowledge of the specific area, prioritizing the use, as far as possible, of validated instruments. On the other hand, in the academic context, it is known that after a certain period of time, knowledge can be decreased in students. Except for the study by Fusco et al. [23], no study performed several measurements in a post-intervention time interval to ensure or, at least, provide information on the permanence and integration of the acquired knowledge. More post-intervention measurements should be introduced in new studies to mitigate this problem.

In most of the studies, we have found similarities with respect to the number of team members, as well as the duration of the ER games, with groups composed of 4 to 7 participants predominating, similar to what is reported in the literature on ER games [4,10,48]. Eukel and Morrell recommend a team size of a maximum of 4 to 5 students to encourage active participation from all members [8]. Regarding the duration of ER games, most studies conducted ER games that did not exceed 60 min, with a minimum duration of 30 min (except in the case of the study by Molina et al. [20], whose duration was 15 min), similar to studies of ER games conducted in other health professionals [4,48].

Finally, it should be noted that studies have only been identified from 5 countries (USA, Australia, Spain, China and Taiwan), which suggests that this educational technology is not yet well implemented in many countries. This could be because in these countries, the universities have enough autonomy to implement new educational technologies. Further research is needed to investigate the factors that encourage the implementation of new educational technologies in certain contexts-countries as opposed to others.

### Limitations

This review has some limitations. The most important is the one mentioned above, referring to the impossibility of being able to perform a meta-analysis, which is the appropriate methodological design to test the effectiveness of an intervention, in this case, the use of ER games in the training of nursing students. Also, the lack of methodologically robust studies available limits this study and its results. In addition to this aspect, we must recognize that an undetermined number of studies may have been left out of the review due to inadequate indexing, as there is sometimes confusion with the term’s gamification, serious games and the like [5,7]. In fact, the lack of standardization and of a clear and unambiguous definition for ER games may influence the exclusion of studies where, according to the authors, ER games were used, either virtually or physically. Finally, some studies evaluated ER games in a set of participants that included nursing students, but not exclusively, which cannot ensure the effectiveness of the educational methodology in this particular population.

## 5. Conclusions

ER games are a recent and growing educational methodology, increasingly used in academia and in the training of nursing students. However, in many countries, this educational technology is not yet implemented. It is therefore necessary to expand its use and the quality of studies in a greater number of contexts and countries. In addition, it is necessary to homogenize and standardize validated instruments to evaluate the effectiveness and real impact of ER games in the area of nursing education. Finally, the usefulness of this type of technology in educational modalities other than the traditional one should be investigated. For example, digital ER games could be a useful technology to achieve student motivation in online educational programs.

## Figures and Tables

**Table 1:** Search strategies in each of the databases.

Database	Date	Search Strategies
Medline (PubMed)	29 December 2023	(“education, nursing”[MeSH Terms] OR “Nursing Education”[All Fields] OR (“education”[All Fields] AND “nursing”[All Fields]) OR “Nursing Education”[All Fields] OR (“educations”[All Fields] AND “nursing”[All Fields])) OR “Nursing Educations”[All Fields]) AND (“Gamification”[MeSH Terms] OR “escape room”[All Fields])
Medline (Ovid)	29 December 2023	Gamification.mp.
		“escape room”.m_titl.
		1 or 2
		Education, Nursing.mp.
		3 and 4
CINHAL (EbscoHOST)	29 December 2023	S1 TX gamification
		S2 TX “escape room” OR TX “scape room” OR TX “escape rooms
		S3 TX “escape room” OR TX “scape room” OR TX “escape rooms”) AND (S1 OR S2)
		S4 TX “nursing education” OR TX “education, nursing”
		S5(TX “nursing education” OR TX “education, nursing”) AND (S3 AND S4)
Scopus (Scopus-Elsevier)	29 December 2023	(ALL (“escape room” OR “escape rooms” OR “scape room”) OR INDEXTERMS (gamification)) AND (INDEXTERMS (“education, nursing” OR “nursing education”))
SCI Expanded (Web of Science)	29 December 2023	((TS = (“gamification”)) OR TS = (“escape room” OR “scape room” OR “escape rooms”)) AND TS = (“education, nursing” OR “nursing education”)

**Table 2:** Characteristics of the included studies.

Author (Year) Country	Design	Themes and Learning Topics	Aim and Main/Secondary Outcomes	Conclusions
Chen et al. (2023) China [15]	Quasi experimental pre-post with CG ^1^	Gerontological Nursing (Safe Medication Care for the Elderly people)	To determine the effects of an intervention educational activity based on an ER ^2^ on nursing students’ learning attitude and the game flow experience after they had received nursing classroom teaching on safe medication use in older adults *Main outcomes*: learnings attitudes and experience of game	During the teaching process of the Gerontological Nursing course, an ER added at the end of classroom teaching can improve nursing students’ learning attitude and also help them to have a good game
Schmuhl et al. (2023) USA [16]	Quasi experimental pre-post	Interprofessional Collaboration and Opioid Use Disorder	To determine the impact of an innovative interprofessional educational activity on healthcare professional students’ learning. The educational activity targeted student knowledge of opioid use disorder and perceptions of working with an interprofessional team while caring for patients with opioid use disorder *Main outcomes*: attitudes about interprofessional collaboration *Secondary outcomes*: perceptions about opioid use disorder	An interprofessional educational experience including both an asynchronous course and virtual synchronous ER can increase participant knowledge around opioid use disorder and may improve student perceptions of working with an interprofessional team and caring for patients with opioid use disorder
Yang et al. (2023) Taiwan [17]	Quasi experimental with CG	Maternity care	To identify the efficiency of ER activities in terms of enhancing nursing students’ retention of maternity-related knowledge and their overall learning performance *Main outcomes*: knowledge about maternity care *Secondary outcomes*: students’ confidence and critical thinking	Maternity ER emerged as an online game-based approach that effectively stimulated nursing students and can serve as a practical resource for engaging in maternity care learning
Hursman et al. (2022) USA [18]	Quasi experimental pre-post	Interprofessional Colaboration	To enhance interprofessional students’ perceptions of their ability to communicate effectively and respectfully, work together to complete a task and to develop knowledge of the unique roles of members of the healthcare team *Main outcomes*: improvement in teamwork (effective communication) *Secondary outcomes*: perceptions and attitudes about gaming	This activity lays the groundwork for collaborative telehealth nursing that students will be exposed to in their future career. Results show the activity helped to build collaboration among team members, including those not in the same physical space. It also showed that virtual ER can be an effective activity to increase interprofessional teamwork perceptions in the online classroom environment and could prove to be useful in other online interprofessional settings
Millsaps et al. (2022) USA [19]	Quasi experimental pre-post	Neurological disorders with a focus on stroke	To promote engagement in undergraduate nursing coursework Main outcomes: knowledge about Stroke	ER experiences can be utilized in the preparation of associate degree nursing education to engage students while also ensuring that students meet key learning objectives
Molina-Torres et al. (2022) Spain [20]	Quasi experimental pre-post with CG	Anatomy	To evaluate the effectiveness of the ER for anatomy-related knowledge retention in nursing and the perceived value of the game *Main outcomes*: knowledge about Anatomy *Secondary outcomes*: satisfaction about gaming	According to the findings, the “Anatomy ER” is a game-based approach that motivates students and constitutes a down-to-earth resource for anatomy learning in healthcare students
Rodríguez-Ferrer et al. (2022) Spain [21]	RCT ^3^	Stigma again Severe Mental Illness	To examine the effect of the Without Memories ER on nursing students’ stigma against Severe Mental Illness *Main outcomes*: modification of stigmatizing attitudes towards severe mental illness	The Without Memories ER can be used as an effective tool to educate and raise awareness about stigmatizing attitudes toward Severe Mental Illness in university students studying health care
Wettergreen et al. (2022) USA [22]	Quasi experimental pre-post	Interprofessional education and the opioid crisis	To evaluate the use of an interprofessional ER activity to increase clinical knowledge related to the opioid crisis. The secondary objective was to evaluate change in attitudes toward interprofessional collaboration *Main outcomes*: knowledge related to the opioid crisis *Secondary outcomes*: attitudes toward interprofessional collaboration	The use of an interprofessional ER as an educational method was effective in increasing some aspects of opioid crisis related knowledge and enhancing attitudes toward interprofessional collaboration. The educational model is applicable to various topics and inter-professional groups
Fusco et al. (2022) USA [23]	RCT	Interprofessional Collaboration Sepsis management and post-operative precautions (hip arthroplasty)	To extend our understanding of ER pedagogical design by investigating the impact of escape room puzzle content on changes in student immediate recall knowledge and demonstration of interprofessional skills during a subsequent interprofessional simulation *Main outcomes*: knowledge of interprofessional collaboration about sepsis *Secondary outcomes*: interprofessional collaborative skills during simulation	ER can be an innovative pedagogical tool that can positively impact immediate recall knowledge and interprofessional collaborative skills of health professions students
Foltz-Ramos et al. (2021) USA [24]	Quasi experimental pre-post with CG	Interprofessional Collaboration	To create and test the use of an interprofessional ER, as a method to improve teamwork, prior to interprofessional simulation *Main outcomes*: improvement of students’ performance in simulation *Secondary outcomes*: attitudes toward interprofessional collaboration	ER can, in a brief period of time, improve teamwork and consequently performance during simulation. Findings support the use of ER in interprofessional education curriculum as a method to promote teamwork
Moore & Campbell (2021) Australia [25]	Quasi experimental pre-post	Interprofessional practice knowledge and competencies	To investigate the utility of an ER coupled with a debriefing workshop as an effective and engaging interprofessional learning activity. To evaluate the impact of the ER on participant knowledge about inter-professional practice and teamwork. To evaluate the impact of the ER through participant reflection on their personal contributions to the team *Main outcomes*: knowledge about interprofessional practice and teamwork and improvement in interprofessional learning activity	The ER intervention added value to the placement curriculum and proved flexible for a heterogeneous student cohort
Gutiérrez-Puertas et al. (2020) Spain [26]	Quasi experimental with CG	Gameful experience Clinical skills	To understand the gameful experience and satisfaction of nursing students in the evaluation of their clinical skills using an ER *Main outcomes*: satisfaction of clinical skills *Secondary outcomes*: experience of game	ER are a useful tool for the evaluation of nursing students compared with using the objective structured clinical evaluation
Morrel & Eukel (2020) USA [27]	Quasi experimental pre-post	Cardiovascular critical care	To evaluate the impact of a cardiovascular ER on student knowledge, as well as to understand student perceptions of the educational innovation *Main outcomes*: knowledge about cardiovascular critical care *Secondary outcomes*: perceptions about gaming	The cardiovascular ER increased student knowledge and was positively received by students. The educational innovation encouraged student engagement in learning, content application, peer communication, and nursing practice skills

^1^ CG: Control group; ^2^ ER: Escape Room games; ^3^ RCT: Randomized Controlled Trial.

**Table 3:** Clinical results of the included studies.

Author (Year)	Instruments	Type of ER Game (Setting) and Time Session (in Minutes)	Size Team (Nursing for Team)	Study Population/Sample (IG ^1^/CG ^2^)	Lost Case (CG/IG)	Pre Mean (SD ^3^) (IG/CG)	Post Mean (SD) (IG/CG)	*p*-Value	Size Effect	Other
Chen et al. (2023) [15]	- LAS ^4^: 23 items, four subscales: learning interest, learning experience, learning habit, and professional recognition. Total range 23–92. Higher scores indicate better learning attitude. - GFEQ ^5^: 19 items, 5 subscales: sense of control, telepresence, distorted sense of time, enjoyable feelings, and being unconscious of irrelevant surroundings. Total range 19–95. Higher scores indicate better game flow experience	Physical ER ^6^ (Geriatric nursing training room) (40)	6–8 (6–8)	84 Nursing students IG = 41 (6 group) CG = 33	None	- LAS: IG = 60.93 (2.33) CG = 61.51(2.32) - CFEQ: IG = 63.27 (2.48)	- LAS: IG = 73.17 (1.67) CG = 61.63 (2.66) - CFEQ: IG = 81.29 (2.49)	- LAS: *p* < 0.001 *t*-test - GFEQ: *p* < 0.001 *t*-test	- LAS: Cohen’s d 5.196 (post-test score) - GFEQ: Cohen’s d 5.253	- LAS (total score 45) for the ER 43.83 (4.49)
Schmuhl et al. (2023) [16]	- ATHCT ^7^ (14 item). Likert (1 = strongly disagree, 2 = disagree, 3 = agree, 4 = strongly agree) - Survey to assess perceptions towards caring for patients with Opioid Use Disorder (11 item) Likert scale (1 = strongly disagree, 2 = disagree, 3 = agree, 4 = strongly agree)	Synchronous virtual ER hosted via zoom breakout rooms (30) Physical ER (simulated emergency room) (90)	Not reported (Team inter professional)	402 health professional students (216 Nursing students) No CG	None	- ATHCT: Performed for 14 items but NP ^8^ for total score. - Opioid Use Disorder: Performed for 11 items but NP for total score	- ATHCT: Performed for 14 items but NP for total score. - Opioid Use Disorder: Performed for 11 items but NP for total score	- ATHCT: *p* < 0.05 *t*-test - Opioid Use Disorder: *p* < 0.05 (7 items) *t*-test	NC ^9^	Following ER, students strongly agreed that their intentions were to change and work collaborative on interprofessional teams
Yang et al. (2023) [17]	- Knowledge test of maternity care: 10 items (maximum score 100 points) - Problem-solving scale: 5 items 5-points Likert scale - Critical thinking questionnaire: 6 items to assess students’ critical thinking abilities, knowledge and confidence	Online game-based ER (50)	6–7 (6–7)	42 Nursing students IG = 21 (Online game-based ER) CG = 21 (online learning without ER)	None	NP	- Knowledge: IG = 30.36 CG = 12.64 - Problem-solving: IG = 28.33 CG = 14.67 - Critical thinking: IG = 31.76 CG = 11.24	- Knowledge: *p* < 0.001 (Mann Whitney U) - Problem-solving: *p* < 0.001 (Mann Whitney U) - Critical thinking: *p* < 0.001 (Mann Whitney U)	NC	
Hursman et al. (2022) [18]	Questionnaire Pre-Post: - Pre-survey 8-item of core competencies for interprofessional collaborative practice - Post-survey 26-item same items more 17 items to evaluate the effectiveness, usefulness of the activity and attitudes toward gaming	Online ER (60)	5–7 (1–2)	176 heath science students (95 Nursing students) No CG	None	NP for 6 items	NP for 6 items	6 items (*p* ˂ 0.001)	NC	
Millsaps et al. (2022) [19]	- 5 questions of knowledge about Stroke	Prequiz (10) Pre-briefing (25) Physical ER (30) Debriefing (25)	4 (4)	Under-graduate ASN ^10^ students (24 students) (12 morning session, and 12 afternoon session) No CG	None	Knowledge: 2.9 (1.06) Median: 3	Knowledge: 3.8 (0.66) Median: 4	*p* = 0.001 for median (Wilconxon)	NC	Not indicated punctuation system
Molina-Torres et al. (2022) [20]	- 10 questions of Knowledge about Anatomy (0–10 points)	Physical ER (University classroom) (15)	4 (4)	248 Nursing students IG = 128 CG = 120	None	NP	Knowledge: IG = 8.94 (0.96) CG = 7.70 (1.25)	Post *p* = 0.001 (Student’s t)	NC	Also measured IG satisfaction using Satisfaction Questionnaire ^11^ (26 questions 1 to 5; higher score higher satisfaction)
Rodríguez-Ferrer et al. (2022) [21]	- Attributional Questionnaire (14-point Likert 1–9; higher score greater number of stigmatizing attitudes toward people with severe mental illness) - Motivation Questionnaire for Cooperative Playful Learning Strategies (Likert scale 1–7)	Web-based ER (60)	4 (4)	316 nursing students randomized IG = 204 (ER no memories) CG = 112 (ER locked In)	IG = 7 CG = 3 Final sample n = 306 IG = 197 CG = 109	Higher scores greater stigma: IG = 47.57 (16.7) CG = 49.56 (16.03)	Higher scores greater stigma expressed: IG = 30.83 (14.79) CG = 49.55 (16.02)	Post *p* ˂ 0.001 (ANOVA)	0.258	
Wettergreen et al. (2022) [22]	- SPICE-R ^12^ Instrument (multiple response and true/false). Likert scale 1–5 points (higher score greater agreement with the statement)	Pre-brief (10) Virtual and Physical ER (60) Debrief (20)	5 (not reported)	80 Heath science students (7 Nursing students) No CG	10 lost	SPICE-R Higher score greater agreement Mean: 4.48	SPICE-R Higher score greater agreement Mean: 4.64	Knowledge: post (*p* ˂ 0.05) (McNemar’s Exact Test)	NC	Pre Knowledge: ^13^ 62.92% Post Knowledge: 74.30%
Fusco et al. (2022) [23]	- ISVS-21 ^14^ - OIPC ^15^ tool: First 10 items: Adequacy of team to a common vision of the situation. Remaining 10 items: Team’s ability to develop a common action plan. For each item, rated a 3-point Likert (1 = inadequate, 2 = more-less adequate, 3 = adequate)	Physical ER (School of Nursing Simulation Center) (30)	4 (2)	233 Nursing and pharmacy students (118 Nursing students) IG = 120 (Simulation) CG = 113 (ER+ simulation)	None	- ISVS-21: IG = 5.3 (0.92) CG = 5.2 (1.0) - OIPC: NP	- ISVS-21: IG = 6.0 (0.72) CG = 5.9 (0.8) - OIPC: Median (IQR ^16^) IG: Items 1–10: 27 (26–28) IG: Items 11–20: 27 (26–28) Total 55 (53–56) CG: Items 1–10: 26 (24–28) CG: Item 11–20: 27 (25–28) Total 53 (49–56)	- ISVS-21: Mean (SD) * IG = 0.72 (0.81) CG = 0.64 (1.0) - OIPC: Items 1–10 *p* < 0.001 Item 11–20 *p* < 0.001 Total *p* < 0.001	Cohen’s d: IG = 0.89 CG = 0.61	
Foltz-Ramos et al. (2021) [24]	- Knowledge Test (10 items multiple choice test) - ISVS-21: 21 items 7-point Likert scale. Items scores are added together and divided by 21 to calculate overall score	Physical ER (Simulation scenario in a Simulation center) (30)	5 (2)	Senior nursing, third-year pharmacy, and second-year physical therapy students IG = 133 (Nursing: 54) ER acute management of sepsis CG = 129 (Nursing: 55) ER general acute care	None	- Knowledge #1: IG = 6.8 (1.9) CG = 6.7 (1.6) - ISVS-21: IG = 5.1 (0.92) CG = 5.2 (0.97)	- Knowledge #2: IG = 7.7 (1.6) CG = 7.3 (1.7) - ISVS-21: IG = 6.0 (0.77) CG = 6.0 (0.82)	- Knowledge #3: *p* = 0.06 - ISVS-21: *p* = 0.70	NC	Three knowledge measures #1, #2, #3
Moore and Campbell (2021) [25]	- Sharif and Nahas’ Questionnaire Adaptation - Knowledge questionnaire: 6 items about knowledge (1 = low–5 = excellent)	Welcome and formal consent (5) Physical ER (55) Comfort break and health care plan development, educational session and evaluation (90)	6 (at least one nursing student)	50 health science students (8 Nursing students) No CG	None	NP	NP	Knowledge difference of pre-post means for 6 questions values *p* ˂ 0.001	NC	
Gutiérrez-Puertas et al. (2020) [26]	- GAMEX ^17^: 7 questions Likert scale (1 = never–5 = always) - Scale for level of satisfaction: scores between 13–52, higher scores indicate higher satisfaction - Practical examination of clinical skill: 10 questions (0, 0.25, 0.5, or 1 point)	Physical ER (30)	5 (5)	237 Nursing students IG = 117 (ER) CG = 120 (OSCE ^18^)	None	NP	Examination of clinical skills IG = 9.59 (0.36) CG = 7.46 (1.36)	*p* ˂ 0.05 (Mann Whitney U)	NC	Results of GAMEX 6 dimensions Mean (SD): - Enjoyment 27.60 (3.02) (range 6–30) - Absorption 22.74 (4.88) (range 6–30) - Creative thinking 15.55 (3.23) (range 4–20) - Activation 16.09 (2.98) (range 4–20) - Absence of negative effects 4.66 (2.32) (range 3–15) - Dominance 13.52 (3.12) (range 4–20)
Morrel and Eukel (2020) [27]	Knowledge questionnaire: - Pre: 10 questions - Post: Same question + perception scale (11 item)	Physical ER (60)	4 (4)	31 Nursing students No CG	2 lost	NP	NP	*p* ˂ 0.05	NC	

^1^ IC: Intervention Group; ^2^ CG: Control Group; ^3^ SD: Standard Deviation; ^4^ LAS: Learning Attitude Scale; ^5^ GFEQ: Game Flow Experience Questionnaire; ^6^ ER: Escape Room games; ^7^ ATCHT: Attitudes Toward Health Care Teams; ^8^ NP: Not Performed; ^9^ NC: Not calculated; ^10^ ASN: Associate of science in nursing; ^11^ Gomez-Urquiza, J.L., Gomez-Salgado, J., Albendín-García, L., Correa-Rodríguez, M., Gonzalez-Jimenez, E., Cañadas-De la Fuente, G.A., 2019. The impact on nursing students’ opinions and motivation of using a “Nursing escape room” as a teaching game: a descriptive study. Nurse Educ. Today 72, 73–76; ^12^ SPICE-R: Student Perceptions of Interprofessional Clinical Education-Revised (SPICE-R 10 questions. The authors did not analyze an overall score but performed a question-by-question analysis. The scores for the 10 questions were summed and divided by 10; ^13^ The average percentage of knowledge has been calculated for the 5 areas (epidemiology, alternatives to opioids, prescription drug monitoring program. Signs of overdose, opioid overdose reversal); ^14^ ISVS-21: Interprofessional Socialization and Valuing Scale; ^15^ OIPC: Observed Interprofessional Collaboration; ^16^ IQR: Interquartile Range; ^17^ GAMEX: Gameful Experience Scale; ^18^ OSCE: objective structured clinical examination; * Mean difference with statistically significant results.

## Data Availability

No new data were created.

## Supplementary Materials

The following supporting information can be downloaded at https://www.mdpi.com/article/10.3390/nursrep14020091/s1, Table S1: Excluded studies from the review, Table S2: Critical appraisal process of the included studies.

## Public Involvement Statement

No public involvement in any aspect of this research.

## Guidelines and Standards Statement

This manuscript was drafted against the Reporting follows the Preferred Reporting Items for Systematic reviews and Meta-Analyses (PRISMA) statement [12].

## References

[1] Kowitlawakul Y., Tan J.J.M., Suebnukarn S., Nguyen H.D., Poo D.C.C., Chai J., Wang W., Devi K. (2022). Utilizing educational technology in enhancing undergraduate nursing students’ engagement and motivation: A scoping review. J. Prof. Nurs..

[2] Jose M.M., Dufrene C. (2014). Educational competencies and technologies for disaster preparedness in undergraduate nursing education: An integrative review. Nurse Educ. Today.

[3] Dahalan F., Alias N., Shaharom M.S.N. (2023). Gamification and Game Based Learning for Vocational Education and Training: A Systematic Literature Review. Educ. Inf. Technol..

[4] Quek L.H., Tan A.J.Q., Sim M.J.J., Ignacio J., Harder N., Lamb A., Chua W.L., Lau S.T., Liaw S.Y. (2024). Educational escape rooms for healthcare students: A systematic review. Nurse Educ. Today.

[5] van Gaalen A.E.J., Brouwer J., Schönrock-Adema J., Bouwkamp-Timmer T., Jaarsma A.D.C., Georgiadis J.R. (2021). Gamification of health professions education: A systematic review. Adv. Health Sci. Educ. Theory Pract..

[6] Li Q., Yin X., Yin W., Dong X., Li Q. (2023). Evaluation of gamification techniques in learning abilities for higher school students using FAHP and EDAS methods. Soft Comput..

[7] Gorbanev I., Agudelo-Londoño S., González R.A., Cortes A., Pomares A., Delgadillo V., Yepes F.J., Muñoz Ó. (2018). A systematic review of serious games in medical education: Quality of evidence and pedagogical strategy. Med. Educ. Online.

[8] Eukel H., Morrell B. (2021). Ensuring Educational Escape-Room Success: The Process of Designing, Piloting, Evaluating, Redesigning, and Re-Evaluating Educational Escape Rooms. Simul. Gaming.

[9] Antón-Solanas I., Rodríguez-Roca B., Urcola-Pardo F., Anguas-Gracia A., Satústegui-Dordá P.J., Echániz-Serrano E., Subirón-Valera A.B. (2022). An evaluation of undergraduate student nurses’ gameful experience whilst playing a digital escape room as part of a FIRST year module: A cross-sectional study. Nurse Educ. Today.

[10] Reinkemeyer E.A., Chrisman M., Patel S.E. (2022). Escape rooms in nursing education: An integrative review of their use, outcomes, and barriers to implementation. Nurse Educ. Today.

[11] Aromataris E., Munn Z. (2020). JBI Manual for Evidence Synthesis.

[12] Page M.J., McKenzie J.E., Bossuyt P.M., Boutron I., Hoffmann T.C., Mulrow C.D., Shamseer L., Tetzlaff J.M., Akl E.A., Brennan S.E. (2021). The PRISMA 2020 statement: An updated guideline for reporting systematic reviews. BMJ.

[13] Munn Z., Stern C., Aromataris E., Lockwood C., Jordan Z. (2018). What kind of systematic review should I conduct? A proposed typology and guidance for systematic reviewers in the medical and health sciences. BMC Med. Res. Methodol..

[14] Rethlefsen M.L., Kirtley S., Waffenschmidt S., Ayala A.P., Moher D., Page M.J., Koffel J.B., PRISMA-S Group (2021). PRISMA-S: An extension to the PRISMA Statement for Reporting Literature Searches in Systematic Reviews. Syst. Rev..

[15] Chen D., Liu F., Zhu C., Tai C., Zhang Y., Wang X. (2023). The effect of an escape room game on college nursing students’ learning attitude and game flow experiences in teaching safe medication care for the elderly: An intervention educational study. BMC Med. Educ..

[16] Schmuhl K.K., Nagel S., Tamburro R., Jewell T.M., Gilbert E., Gonzalez A., Sullivan D.L., Sprague J.E. (2023). Better together: Utilizing an interprofessional course and escape room to educate healthcare students about opioid use disorder. BMC Med. Educ..

[17] Yang C.L., Chang C.Y., Jen H.J. (2023). Facilitating undergraduate students’ problem-solving and critical thinking competence via online escape room learning. Nurse Educ. Pract..

[18] Hursman A., Richter L.M., Frenzel J., Viets Nice J., Monson E. (2022). An online escape room used to support the growth of teamwork in health professions students. J. Interprof. Educ. Pract..

[19] Millsaps E.R., Swihart A.K., Lemar H.B. (2022). Time is brain: Utilizing escape rooms as an alternative educational assignment in undergraduate nursing education. Teach. Learn. Nurs..

[20] Molina-Torres G., Cardona D., Requena M., Rodriguez-Arrastia M., Roman P., Ropero-Padilla C. (2022). The impact of using an “anatomy escape room” on nursing students: A comparative study. Nurse Educ. Today.

[21] Rodriguez-Ferrer J.M., Manzano-León A., Cangas A.J., Aguilar-Parra J.M. (2022). A Web-Based Escape Room to Raise Awareness About Severe Mental Illness Among University Students: Randomized Controlled Trial. JMIR Serious Games.

[22] Wettergreen S.A., Stewart M.P., Huntsberry A.M. (2022). Evaluation of an escape room approach to interprofessional education and the opioid crisis. Curr. Pharm. Teach. Learn..

[23] Fusco N.M., Foltz-Ramos K., Ohtake P.J. (2022). An Interprofessional Escape Room Experience to Improve Knowledge and Collaboration among Health Professions Students. Am. J. Pharm. Educ..

[24] Foltz-Ramos K., Fusco N.M., Paige J.B. (2021). Saving patient x: A quasi-experimental study of teamwork and performance in simulation following an interprofessional escape room. J. Interprof. Care.

[25] Moore L., Campbell N. (2021). Effectiveness of an escape room for undergraduate interprofessional learning: A mixed methods single group pre-post evaluation. BMC Med. Educ..

[26] Gutiérrez-Puertas L., Márquez-Hernández V.V., Román-López P., Rodríguez-Arrastia M.J., Ropero-Padilla C., Molina-Torres G. (2020). Escape Rooms as a Clinical Evaluation Method for Nursing Students. Clin. Simul. Nurs..

[27] Morrell B.L.M., Eukel H.N. (2020). Escape the Generational Gap: A Cardiovascular Escape Room for Nursing Education. J. Nurs. Educ..

[28] Katonai Z., Gupta R., Heuss S., Fehr T., Ebneter M., Maier T., Meier T., Bux D., Thackaberry J., Schneeberger A.R. (2023). Serious Games and Gamification: Health Care Workers’ Experience, Attitudes, and Knowledge. Acad. Psychiatry.

[29] Gentry S.V., Gauthier A., L’Estrade Ehrstrom B., Wortley D., Lilienthal A., Tudor Car L., Dauwels-Okutsu S., Nikolaou C.K., Zary N., Campbell J. (2019). Serious Gaming and Gamification Education in Health Professions: Systematic Review. J. Med. Internet Res..

[30] Akl E.A., Kairouz V.F., Sackett K.M., Erdley W.S., Mustafa R.A., Fiander M., Gabriel C., Schünemann H. (2013). Educational games for health professionals. Cochrane Database Syst. Rev..

[31] Hintze T.D., Samuel N., Braaten B. (2023). A Systematic Review of Escape Room Gaming in Pharmacy Education. Am. J. Pharm. Educ..

[32] Chicca J., Shellenbarger T. (2018). Connecting with Generation Z: Approaches in nursing education. Teach. Learn. Nurs..

[33] Eukel H., Frenzel J., Frazier K., Miller M. (2020). Unlocking Student Engagement: Creation, Adaptation, and Application of an Educational Escape Room across Three Pharmacy Campuses. Simul. Gaming.

[34] Gómez-Urquiza J.L., Gómez-Salgado J., Albendín-García L., Correa-Rodríguez M., González-Jiménez E., Cañadas-De la Fuente G.A. (2019). The impact on nursing students’ opinions and motivation of using a “nursing escape room” as a teaching game: A descriptive study. Nurse Educ. Today.

[35] Shah A.S., Pitt M., Norton L. (2023). ESCAPE the Boring Lecture: Tips and Tricks on Building Puzzles for Medical Education Escape Rooms. J. Med. Educ. Curric. Dev..

[36] Fanning R.M., Gaba D.M. (2007). The role of debriefing in simulation-based learning. Simul. Healthc..

[37] Eppmann R., Bekk M., Klein K. (2018). Gameful Experience in Gamification: Construction and Validation of a Gameful Experience Scale [GAMEX]. J. Interact. Mark..

[38] Gutiérrez-Puertas L., García-Viola A., Márquez-Hernández V.V., Garrido-Molina J.M., Granados-Gámez G., Aguilera-Manrique G. (2021). Guess it (SVUAL): An app designed to help nursing students acquire and retain knowledge about basic and advanced life support techniques. Nurse Educ. Pract..

[39] Kachaturoff M., Caboral-Stevens M., Gee M., Lan V.M. (2020). Effects of peer-mentoring on stress and anxiety levels of undergraduate nursing students: An integrative review. J. Prof. Nurs..

[40] Najjar R.H., Lyman B., Miehl N. (2015). Nursing students’ experiences with high-fidelity simulation. Int. J. Nurs. Educ. Scholarsh..

[41] Reed J.M., Ferdig R.E. (2021). Gaming and anxiety in the nursing simulation lab: A pilot study of an escape room. J. Prof. Nurs..

[42] Hudson A., Franklin K., Edwards T.R., Slivinski A. (2023). Escaping the Silos: Utilization of a Pediatric Trauma Escape Room to Promote Interprofessional Education and Collaboration. J. Trauma Nurs..

[43] Fusco N.M., Foltz-Ramos K., Zhao Y., Ohtake P.J. (2023). Virtual escape room paired with simulation improves health professions students’ readiness to function in interprofessional teams. Curr. Pharm. Teach. Learn..

[44] Abensur Vuillaume L., Laudren G., Bosio A., Thévenot P., Pelaccia T., Chauvin A. (2021). A Didactic Escape Game for Emergency Medicine Aimed at Learning to Work as a Team and Making Diagnoses: Methodology for Game Development. JMIR Serious Games.

[45] Guckian J., Eveson L., May H. (2020). The great escape? The rise of the escape room in medical education. Future Healthc. J..

[46] Dams V., Burger S., Crawford K., Setter R. (2018). Can You Escape? Creating an Escape Room to Facilitate Active Learning. J. Nurses Prof. Dev..

[47] Friedrich C., Teaford H., Taubenheim A., Boland P., Sick B. (2019). Escaping the professional silo: An escape room implemented in an interprofessional education curriculum. J. Interprof. Care.

[48] Veldkamp A., van de Grint L., Knippels M.-C.P.J., van Joolingen W.R. (2020). Escape Education: A Systematic Review on Escape Rooms in Education. Educ. Res. Rev..

