# Supplementary material for: Effectiveness of “Escape Room” Educational Technology in Nurses’ Education: A Systematic Review
Source: Nurs Rep. 2024 May 13;14(2):1193–211. doi: 10.3390/nursrep14020091 (PMC11130902; doi:10.3390/nursrep14020091)
Supplement: Supplementary file 1 [file nursrep-14-00091-s001.zip › nursrep-2938372-supplementary.pdf]

Supplementary Table S1. Excluded studies from the review

ID	Authors	Title	Journal	Year	DOI	Reason for exclusion
84	García-Viola et al	The Influence of Gamification on Decision Making in Nursing Students	The Journal of Nursing Education	2019	10.3928/01484834-20191120-07	Gamification (no Escape Room)
92	Darby et al	Escape Room Relay Race: “Go for the Gold” in Formative Assessment	Journal of Nursing Education	2020	10.3928/01484834-20201020-09	Methodology (Critical appraisal)
37	Daniel, Valko, McAtee & N-Wilfong	Using an Escape Room Modality to Teach Mock Code Essentials	The Journal of Continuing Education in Nursing	2021	10.3928/00220124-20210216-05	Methodology (Critical appraisal)
40	Fitzpatrick, Smith-Brooks, Jones-Parker	Integration of TeamSTEPPS Framework and Escape Room to Improve Teamwork and Collaboration	Journal of Doctoral Nursing Practice	2021	10.1891/JDNP-D-20-00054	Grey Literature (no research article)
42	Frederick & Reed	Operation Outbreak: A Periop 101 Exam Review Escape Room	Simulation & Gaming	2021	10.1177/1046878120948922	Methodology (Critical appraisal)
91	Gabriel et al	Teaching Evidence-Based Sepsis Care: A Sepsis Escape Room	The Journal of Continuing Education in Nursing	2021	10.3928/00220124-20210414-05	Methodology (Critical appraisal)
45	Gutiérrez-Puertas et al	Guess it (SVUAL): An app designed to help nursing students acquire and retain knowledge about basic and advanced life support techniques	Nurse Education in Practice	2021	10.1016/j.nepr.2020.102961	Gamification (no Escape Room)
94	Hwang, Kim & Hwang	The Effect of Game-Based Student Response System (GSRS) on Nursing Education: Focusing on Learning Engagement	Journal of Convergence for Information Technology	2021	10.22156/CS4SMB.2021.11.01.156	Language (Korean)
48	Ma et al	Does theme game-based teaching promote better learning about disaster nursing than scenario simulation: A randomized controlled trial	Nurse Education Today	2021	10.1016/j.nedt.2021.104923	Methodology (Critical appraisal)
52	Putri & Sumartini	Integrating Peer Learning Activities and Problem-Based Learning in Clinical Nursing Education	SAGE Open Nursing	2021	10.1177/23779608211000262	Gamification (no Escape Room)
53	Reed & Ferdig	Gaming and anxiety in the nursing simulation lab: A pilot study of an escape room	Journal of Professional Nursing	2021	10.1016/j.profnurs.2021.01.006	Methodology (Critical appraisal)
57	Sáiz-Manzanares et al	Usefulness of Digital Game-Based Learning in Nursing and Occupational Therapy Degrees: A Comparative Study at the University of Burgos	International Journal of Environmental Research and Public Health	2021	10.3390/ijerph182211757	Gamification (no Escape Room)
67	Wynn	An escape room simulation focused on renal-impairment for prelicensure nursing students	Teaching and Learning in Nursing	2021	10.1016/j.teln.2020.09.006	Methodology (Critical appraisal)
2	Barrickman et al	Development and implementation of a virtual interprofessional escape room to reinforce tobacco cessation concepts	Journal of Interprofessional Education & Practice	2022	10.1016/j.xjep.2022.100511	No Nurses

9	Gu. et al	Effectiveness of a game-based mobile application in educating nursing students on flushing and locking venous catheters with pre-filled saline syringes: A randomized controlled trial	Nurse Education in Practice	2022	10.1016/j.nepr.2021.103260	Gamification (no Escape Room)
23	Pozo-Sánchez, Lampropoulos & López-Belmonte	Comparing Gamification Models in Higher Education Using Face-to-Face and Virtual Escape Rooms	Journal of New Approaches in Educational Research	2022	10.7821/naer.2022.7.1025	No Nurses
29	Wettergreen, Stewart & Huntsberry	Evaluation of an escape room approach to interprofessional education and the opioid crisis	Currents in Pharmacy Teaching and Learning	2022	10.1016/j.cptl.2022.01.021	Methodology (Critical appraisal)
118	Erdogan & Turan	Evaluation of the Effectiveness of Digital Game-Based Learning Given to Nursing Students for the Developmental Care of Infants in Neonatal Intensive Care Unit	CIN Computers Informatics Nursing	2023	10.1097/CIN.0000000000000920	Gamification (no Escape Room)
100	HadaviBavili & İlçioğlu	Artwork in anatomy education: A way to improve undergraduate students' self-efficacy and attitude	Anatomical Science Education	2023	10.1002/ase.2352	Gamification (no Escape Room)
140	Kaynak, Ergün & Karadaş	The effect of crossword puzzle activity used in distance education on nursing students' problem-solving and clinical decision-making skills: A comparative study	Nurse Education in Practice	2023	10.1016/j.nepr.2023.103618	Gamification (no Escape Room)
117	Simsek-Cetinkaya & Cakir	Evaluation of the effectiveness of artificial intelligence assisted interactive screen-based simulation in breast self-examination: An innovative approach in nursing students	Nurse Education Today	2023	10.1016/j.nedt.2023.105857	Gamification (no Escape Room)
99	Hsu et al	An online escape room-based lesson plan to teach new nurses violence de-escalation skills	Nurse Education Today	2023	10.1016/j.nedt.2023.105752	No nursing students
98	Sara & Hunker	An initiative to increase nurse knowledge and decrease postpartum readmissions for preeclampsia	Nursing for Women's Health	2023	10.1016/j.nwh.2023.03.007	Nos nursing students

Supplementary Table S2. Critical appraisal of the included studies (Joanna Briggs Institute tools)

ID	Author; year	Design	% Agreement	Q1	Q2	Q3	Q4	Q5	Q6	Q7	Q8	Q9	Q10	Q11	Q12	Q13
139	Chen et al; 2023 [15]	Quasi experimental	88.8	Yes	Yes	No	Yes	Yes	Yes	Yes	Yes	Yes				
103	Schmuhl et al; 2023 [18]	Quasi experimental	100	Yes	Yes	Yes	Yes	Yes	Yes	Yes	Yes	Yes				
121	Yang et al; 2023 [19]	Quasi experimental	100	Yes	Yes	Yes	Yes	Yes	Yes	Yes	Yes	Yes				
10	Hursman et al; 2022 [20]	Quasi experimental	66,6	Yes	Yes	Yes	Yes	Yes	No	Yes	No	No				
18	Millsaps et al; 2022 [21]	Quasi experimental	66.6	Yes	Yes	Yes	Yes	No	No	Yes	Yes	No				
20	Molina-Torres et al; 2022 [22]	Quasi experimental	88.8	Yes	Yes	No	Yes	Yes	Yes	Yes	Yes	Yes				
25	Rodríguez-Ferrer et al; 2022 [23]	RCT	53.8	No	No	No	No	No	Yes	Yes	Yes	Yes	Yes	Yes	Yes	No
29	Wettergreen et al; 2022 [24]	Quasi experimental	66.6	Yes	Yes	Yes	Yes	Yes	No	Yes	No	No				
93	Fusco et al; 2022 [25]	RCT	92.3	Yes	Yes	Yes	No	Yes	Yes	Yes	Yes	Yes	Yes	Yes	Yes	Yes
41	Foltz-Ramos et al; 2021 [26]	Quasi experimental	66.6	No	No	No	Yes	Yes	Yes	Yes	Yes	Yes				
50	Moore & Campbell; 2021 [27]	Quasi experimental	88.8	Yes	Yes	Yes	Yes	Yes	Yes	Yes	Yes	No				
72	Gutiérrez-Puertas et al; 2020 [28]	Quasi experimental	100	Yes	Yes	Yes	Yes	Yes	Yes	Yes	Yes	Yes				
78	Morrel & Eukel; 2020 [29]	Quasi experimental	77.7	Yes	Yes	Yes	Yes	Yes	No	Yes	No	Yes				

Quasi-experimental (Q1: Is it clear in the study what is the 'cause' and what is the 'effect' (i.e. there is no confusion about which variable comes first)?; Q2: Were the participants included in any comparisons similar?; Q3: Were the participants included in any comparisons receiving similar treatment/care, other than the exposure or intervention of interest?; Q4: Was there a control group?; Q5: Were there multiple measurements of the outcome both pre and post the intervention/exposure?; Q6: Was follow up complete and if not, were differences between groups in terms of their follow up adequately described and analyzed?; Q7: Were the outcomes of participants included in any comparisons measured in the same way?; Q8: Were outcomes measured in a reliable way?; Q9: Was appropriate statistical analysis used?).

RCT (Q1: Was true randomization used for assignment of participants to treatment groups?; Q2: Was allocation to treatment groups concealed?; Q3: Were treatment groups similar at the baseline?; Q4: Were participants blind to treatment assignment?; Q5: Were those delivering treatment blind to treatment assignment?; Q6: Were outcomes assessors blind to treatment assignment?; Q7: Were treatment groups treated identically other than the intervention of interest?; Q8: Was follow up complete and if not, were differences between groups in terms of their follow up adequately described and analyzed?; Q9: Were participants analyzed in the groups to which they were randomized?; Q10: Were outcomes measured in the same way for treatment groups?; Q11: Were outcomes measured in a reliable way?; Q12: Was appropriate statistical analysis used?; Q13: Was the trial design appropriate, and any deviations from the standard RCT design (individual randomization, parallel groups) accounted for in the conduct and analysis of the trial?).
